# Noncoding variations in *Cyp24a1 *gene are associated with Klotho‐mediated aging phenotypes in different strains of mice

**DOI:** 10.1111/acel.12949

**Published:** 2019-03-28

**Authors:** Amit Singh, Anjali Verma, Michelle A. Sallin, Florian Lang, Ranjan Sen, Jyoti Misra Sen

**Affiliations:** ^1^ National Institute on Aging National Institutes of Health Baltimore Maryland; ^2^ Institute of Physiology Eberhard Karls University of Tübingen Tübingen Germany; ^3^ Department of Medicine The Johns Hopkins University School of Medicine Baltimore Maryland

**Keywords:** aging, genetic background, klotho‐hypomorphic allele, vitamin D metabolism

## Abstract

In mutant mice, reduced levels of Klotho promoted high levels of active vitamin D in the serum. Genetic or dietary manipulations that diminished active vitamin D alleviated aging‐related phenotypes caused by Klotho down‐regulation. The hypomorphic Klotho [*kl/kl*] allele that decreases Klotho expression in C3H, BALB/c, 129, and C57BL/6 genetic backgrounds substantially increases 1,25(OH)2D3 levels in the sera of susceptible C3H, BALB/c, and 129, but not C57BL/6 mice. This may be attributed to increased basal expression of *Cyp24a1 *in C57BL/6 mice, which promotes inactivation of 1,25(OH)2D3. Decreased expression of *Cyp24a1 *in susceptible strains was associated with genetic alterations in noncoding regions of *Cyp24a1 *gene, which were strongly reminiscent of super‐enhancers that regulate gene expression. These observations suggest that higher basal expression of an enzyme required for catabolizing vitamin D renders *B6‐kl/kl *mice less susceptible to changes in Klotho expression, providing a plausible explanation for the lack of aging phenotypes on C57BL/6 strain.

## 1 INTRODUCTION

The *Klotho *gene encodes α‐Klotho, a protein with sequence homology to β‐glucuronidase enzymes (reviewed in Dalton, Xie, An, & Huang, 2017). The hypomorphic *Klotho *(*kl/kl*) mutation displayed premature aging phenotypes in C3H‐*kl/kl *and BALB/c‐*kl/kl *mice (Kuro‐o et al., 1997). Deletion of the *Klotho *gene in 129 genetic background has also been shown to result in severe aging‐related phenotypes (University of California, Davis, Mutant Mouse Regional Resource Centers). By contrast, the *kl/kl *mutation on the C57BL/6 genetic background displayed normal lifespan with reduced aging‐related phenotypes (Phelps, Pettan‐Brewer, Ladiges, & Yablonka‐Reuveni, 2013). These observations indicate that genetic differences between inbred mouse strains play a role in aging‐related outcomes of the *kl/kl *mutation. Klotho is primarily expressed in the kidney where it has been shown to regulate vitamin D metabolism (Kuro‐o et al., 1997). Decreased Klotho expression in C3H‐*kl/kl *mice correlates with increased serum levels of FGF‐23, phosphate, calcium, and vitamin D3 [1,25(OH)2D3] (Kuro‐o, 2006). Increased levels of active 1α,25‐dihydroxy vitamin D3 [1α,25(OH)2D3] were shown to be a major cause of the aging‐related phenotypes in aging‐susceptible strains of Klotho mutant mice (Leibrock, Voelkl, Kuro, Lang, & Lang, 2016). However, the basis for the difference in age‐related phenotypes in *kl/kl *mutant mice on different genetic backgrounds remains unknown. To address the difference in aging‐related phenotypes between susceptible [C3H, 129, and BALB/c] and nonsusceptible [C57BL/6] strains, we assayed changes in metabolic pathways that have been associated with Klotho function. In particular, we addressed the expression of *Cyp24a1 *and *Cyp27b1 *genes whose products are known to regulate the levels of vitamin D3 in the serum (Christakos, Ajibade, Dhawan, Fechner, & Mady, 2010).

## 2 RESULTS AND DISCUSSION

**Figure 1:**
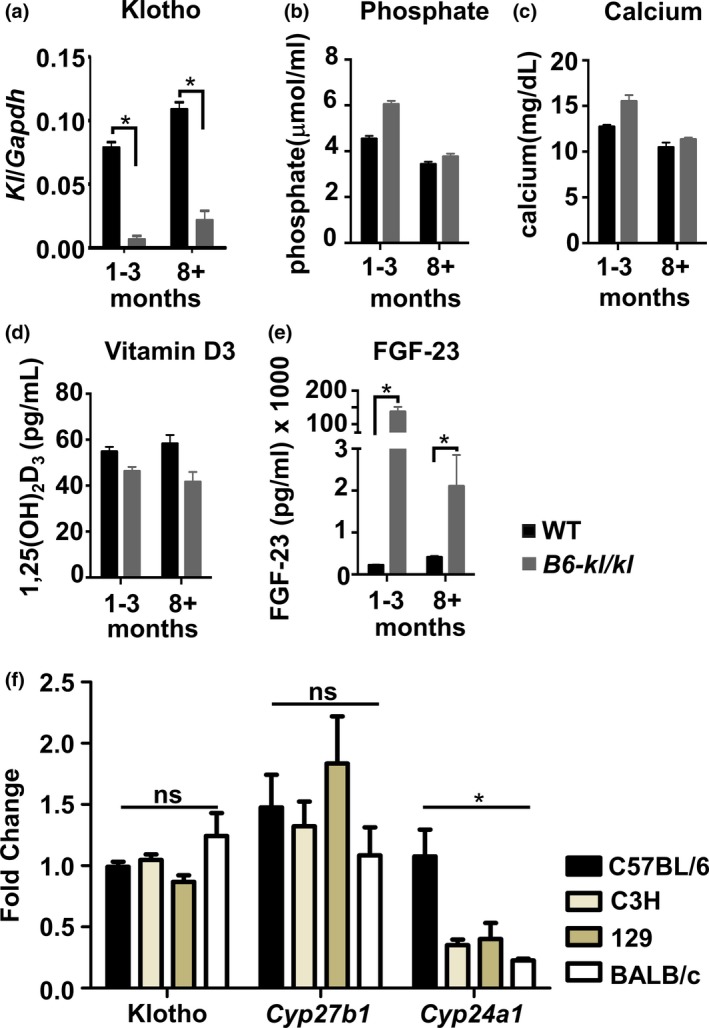
Description of B6‐*kl/kl *mice. (a) *Klotho *mRNA expression in B6 and B6‐*kl/kl *kidney normalized to *Gapdh*. (b) phosphate, (c) calcium, (d) vitamin D3, and (e) FGF‐23 levels in serum of B6 and B6‐*kl/kl *mice at different ages. C57BL/6 (young *n* = 6, old *n* = 7) and B6‐*kl/kl *(young *n* = 8, old *n* = 7)*. *(f) Expression of Klotho, *Cyp27b1, *and *Cyp24a1 *in kidney of indicated mouse strains. C57BL/6 (*n* = 11), C3H (*n* = 8), 129 (*n* = 6), and BALB/c (*n* = 2) mice. *p < 0.05–0.001

Despite significantly reduced *Klotho *expression (Figure 1a), B6‐*kl/kl *mice showed no significant increases in serum phosphate, calcium, and vitamin D3 levels (Figure 1b–d) on a standard rodent diet (Supporting Information Table S1). Accordingly, the increase of FGF‐23 levels following Klotho decrease (Figure 1e) was also less dramatic in B6‐*kl/kl *mice than in the susceptible strains (Leibrock et al., 2016). These data provide a plausible explanation for the lack of premature aging phenotypes in B6‐*kl/kl *mice.

To further probe the absence of increased vitamin D levels and aging‐related phenotypes, we interrogated expression of Cyp27b1 and Cyp24a1, which are involved in controlling serum levels of active vitamin D3, 1,25(OH)2D3 (Christakos et al., 2010). To determine basal expression, we assayed mRNA for *Cyp27b1 *and *Cyp24a1 *in kidneys from C57BL/6, C3H, 129, and BALB/c mice (Figure 1f). Whereas, basal levels of *Klotho *and *Cyp27b1 *expression were comparable in all strains of mice, we found that basal expression of *Cyp24a1 *was significantly higher in kidneys of C57BL/6 mice compared to susceptible strains (Figure 1f). Because Klotho/FGF‐23 heterodimer has been implicated in regulating vitamin D metabolism via *Cyp27b1 *and *Cyp24a1*, these observations suggest that lower basal level of *Cyp24a1 *expression in susceptible strains may render those mice more reliant on Klotho/FGF‐23 and FGFR‐axis to maintain healthy levels of serum vitamin D3. We propose that high basal expression of *Cyp24a1 *makes C57BL/6 mice less sensitive to decreased *Klotho *expression and contributes to abrogation of Klotho‐dependent aging‐related phenotypes in B6‐*kl/kl *mice.

**Figure 2:**
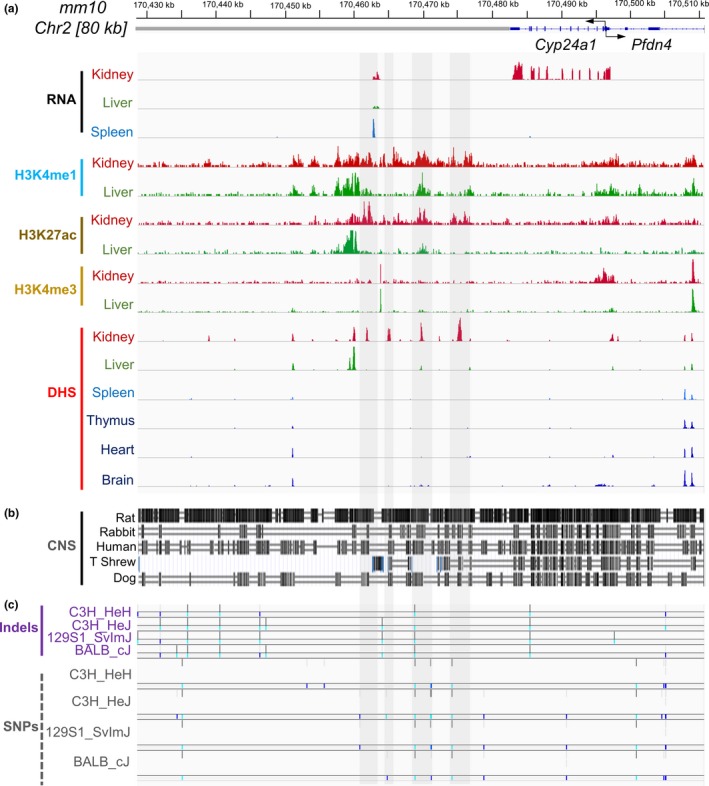
Genetics and epigenetics of the *Cyp24a1 *gene locus. (a) A portion of chromosome 2 containing the *Cyp24a1 *gene and flanking sequences, top row; RNA, RNA‐seq tracks from indicated tissues; H3K4me1, H3K27ac, and H3K4me3 refer to locations of these forms of modified histones obtained from chromatin immunoprecipitation and sequencing (ChIP‐seq) analysis of indicated tissues, middle rows; DHS: DNase 1 hypersensitive site analysis of *Cyp24a1 *flanking sequences in indicated tissues, bottom row. (b) Comparison of tracks for conserved noncoding sequences for indicated species with reference C57BL/6 genome. (c) Single nucleotide polymorphism (SNPs) and insertions and deletions (Indels) in various mouse strains compared to reference C57BL/6 genome. Regions of possible regulatory function based on epigenetic marks are indicated by highlighted areas in a–c

To uncover regulatory mechanisms that alter expression of *Cyp24a1 *in susceptible mouse strains compared to C57BL/6, we examined chromatin state and genetic variations near the *Cyp24a1 *gene. Kidney‐specific expression of *Cyp24a1 *was evident in RNA sequencing profiles (Figure 2a, tracks labeled RNA). The 20 kb region immediately 3′ of *Cyp24a1 *contained four prominent DNase 1 hypersensitive sites (DHS) specific to kidney samples and a fifth site that was shared with liver (Figure 2a, tracks labeled DHS). The known relationship between DHS and transcriptional enhancers suggested that this region may regulate *Cyp24a1 *expression. As another measure of regulatory potential, we examined epigenetic histone modifications associated with transcriptional enhancers. This entire region was selectively marked by enhancer‐associated histone H3 acetylated at lysine 27 (H3K27ac) and histone H3 mono‐methylated at lysine 4 (H3K4me1) only in kidney samples. The broad distribution of these marks was reminiscent of super‐enhancers (Whyte et al., 2013; Witte, O'Shea, & Vahedi, 2015). The close tissue‐specific correlation between expression of *Cyp24a1 *and presence of H3K27ac‐ and H3K4me1‐marked modified DHS suggests that this cluster of DHS regulates kidney‐specific expression of *Cyp24a1 *gene.

Sequences surrounding these regions of chromatin modifications and DHS were also found to be conserved in other mammalian species, including humans (Figure 2b, tracks labeled CNS [conserved noncoding sequence]), suggesting common regulatory pathway across species. Remarkably, the same region contained multiple genetic variations/deletions in the Klotho‐susceptible strains compared to C57BL/6 (Figure 2c, tracks labeled Indels, SNPs). We propose that these noncoding genetic variations in the putative *Cyp24a1 *super‐enhancer lead to reduced basal expression of *Cyp24a1 *in susceptible strains making them more reliant on *Klotho *for regulation of *Cyp24a1 *to maintain healthy levels of serum vitamin D3.

## CONFLICT OF INTEREST

None declared.

## AUTHOR CONTRIBUTIONS

JMS designed and supervised the study; AV and MS performed the experiments; AS and RS did the genetic analysis of kidney‐specific variations in *Cyp24a1 *gene. JMS and RS wrote the paper and all authors read and commented on the manuscript.
