# Supplementary material for: Noncoding variations in Cyp24a1 gene are associated with Klotho‐mediated aging phenotypes in different strains of mice
Source: Aging Cell. 2019 Mar 28;18(3):e12949. doi: 10.1111/acel.12949 (PMC6516175; doi:10.1111/acel.12949)
Supplement: Supplementary file 1 [file ACEL-18-e12949-s001.pdf]

Table S1: composition of standard rodent diet

2018SX

ENVIGO

Teklad Global 18% Protein Extruded Rodent Diet (Sterilizable)

Product Description- 2018SX is a fixed formula, autoclavable extruded diet manufactured with high quality ingredients and designed to support gestation, lactation, and growth of rodents. 2018SX does not contain alfalfa, thus lowering the occurrence of natural phytoestrogens. Typical isoflavone concentrations (daidzein + genistein aglycone equivalents) range from 150 to 250 mg/kg. Exclusion of alfalfa reduces chlorophyll, improving optical imaging clarity. Absence of animal protein and fish meal minimizes the presence of nitrosamines. 2018SX is supplemented with additional vitamins to ensure nutritional adequacy after autoclaving and the extruded form helps reduce diet clumping and hardness.

Ingredients (in descending order of inclusion)- Ground wheat, ground corn, wheat middlings, dehulled soybean meal, corn gluten meal, soybean oil, calcium carbonate, dicalcium phosphate, brewers dried yeast, iodized salt, L-lysine, DL-methionine, calcium propionate, choline chloride, kaolin, menadione sodium bisulfite complex (source of vitamin K activity), magnesium oxide, vitamin E acetate, calcium pantothenate, thiamin mononitrate, manganous oxide, niacin, ferrous sulfate, zinc oxide, riboflavin, vitamin A acetate, pyridoxine hydrochloride, copper sulfate, vitamin B_{12} supplement, folic acid, calcium iodate, biotin, vitamin D_3 supplement, cobalt carbonate.

Standard Product Form: ***Extruded***

Macronutrients		
Crude Protein	%	18.6
Fat (ether extract) [a]	%	6.2
Fat (acid hydrolysis) [a]	%	7.0
Carbohydrate (available) [b]	%	44.2
Crude Fiber	%	3.5
Neutral Detergent Fiber [c]	%	14.7
Ash	%	5.3
Energy Density [d]	kcal/g (kJ/g)	3.1 (13.0)
Calories from Protein	%	24
Calories from Fat	%	18
Calories from Carbohydrate	%	58
**Minerals**		
Calcium	%	1.0
Phosphorus	%	0.7
Non-Phytate Phosphorus	%	0.4
Sodium	%	0.2
Potassium	%	0.6
Chloride	%	0.4
Magnesium	%	0.2
Zinc	mg/kg	70
Manganese	mg/kg	100
Copper	mg/kg	15
Iodine	mg/kg	6
Iron	mg/kg	200
Selenium	mg/kg	0.23
**Amino Acids**		
Aspartic Acid	%	1.4
Glutamic Acid	%	3.4
Alanine	%	1.1
Glycine	%	0.8
Threonine	%	0.7
Proline	%	1.6
Serine	%	1.1
Leucine	%	1.8
Isoleucine	%	0.8
Valine	%	0.9
Phenylalanine	%	1.0
Tyrosine	%	0.6
Methionine	%	0.6
Cystine	%	0.3
Lysine	%	1.1
Histidine	%	0.4
Arginine	%	1.0
Tryptophan	%	0.2

Vitamins		
Vitamin A [e, f]	IU/g	30.0
Vitamin D_3 [e, g]	IU/g	2.0
Vitamin E	IU/kg	135
Vitamin K_3 (menadione)	mg/kg	100
Vitamin B_1 (thiamin)	mg/kg	117
Vitamin B_2 (riboflavin)	mg/kg	27
Niacin (nicotinic acid)	mg/kg	115
Vitamin B_6 (pyridoxine)	mg/kg	26
Pantothenic Acid	mg/kg	140
Vitamin B_{12} (cyanocobalamin)	mg/kg	0.15
Biotin	mg/kg	0.90
Folate	mg/kg	9
Choline	mg/kg	1200
**Fatty Acids**		
C16:0 Palmitic	%	0.7
C18:0 Stearic	%	0.2
C18:1ω9 Oleic	%	1.2
C18:2ω6 Linoleic	%	3.1
C18:3ω3 Linolenic	%	0.3
Total Saturated	%	0.9
Total Monounsaturated	%	1.3
Total Polyunsaturated	%	3.4
**Other**		
Cholesterol	mg/kg	--

[a] Ether extract is used to measure fat in pelleted diets, while an acid hydrolysis method is required to recover fat in extruded diets. Compared to ether extract, the fat value for acid hydrolysis will be approximately 1% point higher.

[b] Carbohydrate (available) is calculated by subtracting neutral detergent fiber from total carbohydrates.

[c] Neutral detergent fiber is an estimate of insoluble fiber, including cellulose, hemicellulose, and lignin. Crude fiber methodology underestimates total fiber.

[d] Energy density is a calculated estimate of *metabolizable energy* based on the Atwater factors assigning 4 kcal/g to protein, 9 kcal/g to fat, and 4 kcal/g to available carbohydrate.

[e] Indicates added amount but does not account for contribution from other ingredients.

[f] 1 IU vitamin A = 0.3 μg retinol

[g] 1 IU vitamin D = 25 ng cholecalciferol

For nutrients not listed, insufficient data is available to quantify.

Nutrient data represent the best information available, calculated from published values and direct analytical testing of raw materials and finished product. Nutrient values may vary due to the natural variations in the ingredients, analysis, and effects of processing.

Teklad Diets are designed and manufactured for research purposes only.

SGS

© 2015 Envigo

0915
