# Supplementary material for: Noncoding variations in Cyp24a1 gene are associated with Klotho‐mediated aging phenotypes in different strains of mice
Source: Aging Cell. 2019 Mar 28;18(3):e12949. doi: 10.1111/acel.12949 (PMC6516175; doi:10.1111/acel.12949)
Supplement: Supplementary file 2 [file ACEL-18-e12949-s002.docx]

**Supporting Information Experimental Procedures** *Ethics Statement*

Investigation has been conducted in accordance with the ethical standards and according to the Declaration of Helsinki and according to national and international guidelines and has been approved by authors’ institutional review board.

*Mice*

Klotho-hypomorphic *kl/kl* mice on C3H/J background [C3H-*kl/kl*] (Kuro-o *et al.* 1997) were backcrossed for 10 generations to C57BL/6J mice obtained from The Jackson Laboratory. B6-*kl/kl* mice were screened by conventional PCR immediately after weaning using the primer forward: 5’- tggagattggaagtggac-3’ together with the primers reverse: 5’-caaggaccagttcatcatcg-3’ and reverse: 5’-ttaaggactcctgcatctgc-3’ for the klotho hypomorphic and for the WT genotype, respectively. All mice were bred and maintained in an animal facility at the National Institute of Aging (NIA) in a 12-hour light-dark cycle and fed ad libitum with a diet containing 0.7% of phosphorus (0.4% nonphytate phosphorus). The studies were carried out in accordance with the recommendations in the Guide for the Care and Use of Laboratory Animals. The protocol was approved by the Animal Care and Use Committee of the NIA Intramural Research Program, NIH.

*C57BL/6 background genotyping*

Klotho-hypomorphic mice on C57BL/6/C3H mixed background were backcrossed to C57BL/6J mice for more than 10 generations to generate B6-*kl/kl* mice. Tail samples from C57BL/6 and B6- *kl/kl* mice animals were sent to the Animal Molecular Diagnostics Laboratory at Frederick National Laboratory for Cancer Research to verify congenicity with C57BL/6 strain. Briefly, 1cm tails from 10 mutant mice were digested and DNA extracted using DNeasy® Blood & Tissue Kit (Qiagen). DNA samples were then quantified using a nanodrop (SPECTROstar Nano) and screened for 96 C57BL/6 microsatellite markers distributed over 19 chromosomes using an ABI 3130 Genetic Analyzer.

*Serum chemistry*

Serum phosphate and serum calcium were determined through colorimetric assays (Abcam). Briefly, freshly collected serum and urine samples were diluted 1:300 and 1:10, respectively, and mixed together with detection reagents for 10-30min at room temperature. Absorbances were measured in a SinergyTM H1 microplate reader (BioTEK). Serum FGF-23 concentration was determined using an ELISA kit (Abcam) and serum 1,25(OH)2D3 was determined using a competitive EIA kit (LifeSpan BioSciences) following manufacturer’s instructions. Serial dilutions with known concentrations were used to establish standard curves.

*Gene Expression Analysis*

Kidneys from C3H, 129, BALB/c, and C57BL/6 mice were collected for RNA extraction and qPCR analysis. Briefly, kidneys were disrupted and homogenized using a TissueRuptor II, and RNA was extracted from lysates using the RNeasy mini kit (Qiagen). DNA was eliminated from the samples by incubating with DNase (Qiagen). First-strand cDNA synthesis was performed by using 1μg of total RNA together with oligo(dT)12–18 and the SuperScript II Reverse Transcriptase (Invitrogen), accordingly to the manufacturer's instructions. Quantification of *Klotho*, *Cyp27b1* and *Cyp24a1* mRNA expression was conducted using real-time qPCR performed on a ViiA™ 7 Real-Time PCR System. Primers were designed to amplify specific amplicons of *kl* (F:5’-gacgctcgggtacctggtt-3’; R:5’-ggcggaaagaggtgttgtagag-3’), *Cyp27b1* (F:5’-gatgtttgcctttgcccaga-3’; R:5’- ggcatatcctcctcaggctt-3’), *Cyp24a1* (F:5’-acgctacccaaaggaacagt-3’; R:5’-gacgccaaatgggagatgag-3’) and*gapdh*(F:5’-gtcgtggagtctactggtgtc; R:5’-cagaaggggcggagatgatg-3’) genes. Each cDNA sample was diluted 5-fold and then 5μL of dilutions added to 5pmol of each primer and SYBR Green Master (Roche). The cycling parameters were as follows: 10 min at 95°C, followed by 40 cycles of 15s at 95°C and 1min at 60°C. Quantification of gene expression was performed by the 2^-ΔCt method using *gapdh* as the normalizer gene. Each sample was quantified in triplicate and primer amplification efficiencies were calculated and validated with the standard curves obtained through the amplification of cDNA serial dilution.

*Epigenetic analysis*

Expression [RNA-seq] and chromatin status of the *Cyp24a1* gene was obtained from the ENCODE database [https://[www.encodeproject.org/](http://www.encodeproject.org/)]. Conserved noncoding sequences [CNS] for rat, rabbit, human, tree shrew and dog were obtained from “Vertebrate Multiz Alignment & Conservation (100 Species).” SNP’s and INDEL’s: data were downloaded from [ftp://ftpmouse.sanger.ac.uk/current_snps/strain_specific_vcfs/]. RNA seq, histone ChIP-Seq,

DHS and genetic variation results were shown using Integrative Genomics Viewer (Broad Institute). CNS tracks are shown from UCSC Genome Browser.

*Statistical Analysis*

Data are presented as mean±standard error mean. Significance was determined using multiple *t* tests with GraphPad Prism 6.0 (GraphPad) software.
